# *Landau damping* effects in the synchronization of conformist and contrarian oscillators

**DOI:** 10.1038/srep18235

**Published:** 2015-12-14

**Authors:** Tian Qiu, Yue Zhang, Jie Liu, Hongjie Bi, S. Boccaletti, Zonghua Liu, Shuguang Guan

**Affiliations:** 1 Department of Physics, East China Normal University, Shanghai, 200241, China; 2 CNR-Institute of Complex Systems, Via Madonna del Piano, 10, 50019 Sesto Fiorentino, Florence, Italy; 3 The Embassy of Italy in Tel Aviv, 25 Hamered street, 68125 Tel Aviv, Israel; 4 State Key Laboratory of Theoretical Physics, Institute of Theoretical Physics, Chinese Academy of Sciences, Beijing 100190, China

## Abstract

Two decades ago, a phenomenon resembling Landau damping was described in the synchronization of globally coupled oscillators: the evidence of a regime where the order parameter decays when linear theory predicts neutral stability for the incoherent state. We here show that such an effect is far more generic, as soon as phase oscillators couple to their mean field according to their natural frequencies, being then grouped into two distinct populations of *conformists* and *contrarians*. We report the analytical solution of this latter situation, which allows determining the critical coupling strength and the stability of the incoherent state, together with extensive numerical simulations that fully support all theoretical predictions. The relevance of our results is discussed in relationship to collective phenomena occurring in social and economical systems.

In the forties, the Soviet physicist Lev Davidovich Landau (the 1962 Nobel Laureate for his theory on superfluidity) predicted the damping (the exponential decrease as a function of time) of electrostatic charge waves in a collision-less plasma^1^,^2^. After almost two decades of controversy, the “Landau damping” (LD) was eventually verified experimentally^3^, and it was even argued that similar phenomena could take place in galactic dynamics^4^. Mathematically, LD is entirely due to the occurrence of fake eigenvalues caused by analytic continuation, which lead to an exponential decay of the electric field even when the density perturbation does not.

More recently, Strogatz *et al.* investigated the synchronization transition in ensembles of coupled oscillators^5^, and found a regime (below the synchronization threshold, and for which linear theory foretells neutral stability) where the relaxation to the incoherent state is indeed exponential, with a decaying mechanism remarkably similar to LD.

In this paper, we report on theoretical analysis and numerical simulations that demonstrate how the presence of LD is, actually, far more general in the synchronization route of globally coupled oscillators. Without lack of generality, we start by considering a frequency-weighted Kuramoto^6^ model of *N* phase oscillators, in which units are coupled to the mean field according to their natural frequencies:


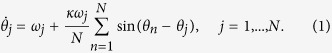


Here *θ*_*j*_ (*ω*_*j*_) is the instantaneous phase (the natural frequency) of the *j*th oscillator, dot denotes a temporal derivative, and *κ* > 0 is a global coupling strength parameter. The set {*ω*_*j*_} of natural frequencies is drawn from a given frequency distribution (FD) *g*(*ω*) with, in general, both a positive and a negative domain. Eq. (1) belongs to the class of the so-called generalized Kuramoto models^6^,^7^,^8^,^9^,^10^,^11^. At variance with refs 12,13, the effective coupling *κω*_*j*_ can be here either positive or negative (depending on the sign of the natural frequency *ω*_*j*_), reflecting the fact that the interactions among individuals can inherently be repulsive in real systems^14^,^15^,^16^,^17^. For instance, it is well known that both excitatory and inhibitory couplings characterize the interaction structure of neural ensembles^18^,^19^, as well as similar kinds of coupling can also be found in social interactions.

Eq. (1) is fully analytically solvable, and we here focus on unveiling the details of several novel phenomena of LD, which characterize the transition to synchronization in such systems. In its mean-field form, Eq. (1) can be written as


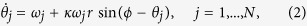


where *r* and *ϕ* are order parameters defined by


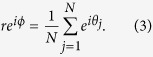


Here oscillators can be, in general, grouped into two populations, according to their effective coupling: those with positive *ω*_*j*_ will behave like *conformists* attempting to follow the global rhythm of the system, whereas those with negative *ω*_*j*_ will tend to act as *contrarians*, i.e. they will always try opposing the system’s global beat^15^,^16^,^17^. Let us then analyze the synchronization transition in Eq. (1) in the presence of a Lorentzian FD


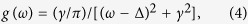


where Δ and 2*γ* are the central frequency and the width at half maximum, respectively, with Δ actually controlling the proportion of conformists to contrarians in the ensemble.

In this work, numerical integrations of coupled ordinary differential equations are performed by the fourth-order Runge-Kutta method with time step 0.01. The initial conditions for the phase variables are random. Typically, the total number of oscillators is *N* = 10000. For simplicity, the network is supposed to be globally coupled. To test if hysteresis exists in the synchronization transitions, we study both the forward and the backward transitions in an adiabatic way. For each control parameter, the order parameter is averaged in a time window after the transient stage. Such numerical schemes are adopted throughout this paper.

## Results

### Linear stability analysis

In the thermodynamic limit, i.e., *N* → ∞, a density function *ρ*(*θ*, *ω*, *t*) can be defined, where *ρ dθ* denotes the fraction of oscillators with frequency *ω* whose phases have values between *θ* and *θ* + *dθ* at time *t*. *ρ* satisfies the normalization condition 
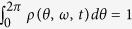
 for all *ω*, and all *t*. The evolution of *ρ* is governed by the continuity equation


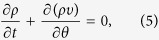


where the velocity *υ* is given by Eq. (2) as *υ* = *ω* + *κωr*sin(*ϕ* − *θ*). On its turn, the order parameter can be expressed as


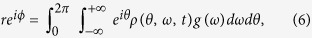


and therefore Eq. (5) can be rewritten as


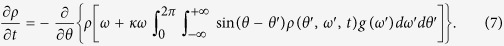


For the incoherent state, *ρ*_0_(*θ*, *ω*, *t*) = 1/(2*π*). A small perturbation from that state, i.e.


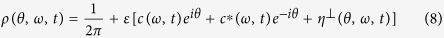


can be considered, where 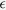 ≪ 1, and *η*^⊥^(*θ*, *ω*, *t*) represents the higher Fourier harmonics. Substituting Eq. (8) into Eq. (7), one gets the linearized characteristic equation


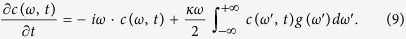


Equation (9) has both a continuous and a discrete spectrum. Following the analysis of ref. 7, the continuous spectrum is the set {−*iω* : *ω* ∈ *Support*(*g*)}, which is the whole imaginary axis for the Lorentzian FD. As for the discrete spectrum that determines the stability of *c*(*ω*, *t*), one has to seek solutions of the form *c*(*ω*, *t*) = *b*(*ω*)*e*^*λt*^, where *λ* is independent of *ω*. Then, Eq. (9) becomes


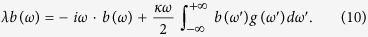


Equation (10) can be solved in a self-consistent way. Precisely, let 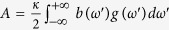. Then, *b*(*ω*) can be solved from Eq. (10) as: *b*(*ω*) = *ωA*/(*λ* + *iω*). By substituting this back into the expression of *A*, we ultimately obtains the critical equation


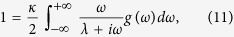


which relates *κ* with the eigenvalue *λ*. Substituting Eq. (4) into Eq. (11), one gets:


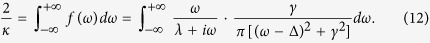


Equivalently,


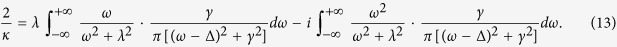


From Eq. (13), one sees that if *λ* is real, the result of the first integral is real, while the second integral contributes a pure number (its integrand is non-negative except at the point *ω* = 0). Then, Eq. (13) cannot be satisfied because the left-hand side is real. The consequence is that the eigenvalue *λ* must be complex, and this is essentially different from the case of the classical Kuramoto model, where the discrete eigenvalues are proven to be real and positive^7^.

The direct integration of Eq. (12) turns out to be tedious. The alternative is looking for a contour integration in the complex plane. For this purpose, one can set *λ* = *a* + *ib* (*a*, *b* ∈ *R*, and *b* ≠ 0) and discuss three distinct cases, corresponding to *a* > 0, *a* = 0, and *a* < 0, respectively. The detailed processes are as follows.

1. *a* > 0. In this case, *f*(*ω*) has two poles *ω*_1_ =  − *b* + *ia* and *ω*_2_ = Δ + *iγ* in the upper half complex plane; and one pole *ω*_3_ = Δ − *iγ* in the lower half complex plane. So the integral in Eq. (12) can be conveniently done by choosing a contour in the lower half complex plane. Accordingly, Eq. (12) becomes:
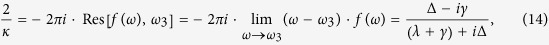
where Res means the residue. From this equation we explicitly get the closed form of the eigenvalue as
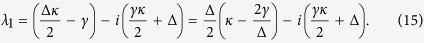
The real part of *λ*_1_ determines the stability of the incoherent state, i.e., when Re[*λ*_1_] changes from negative to positive, the incoherent state loses its stability. Notice that *a* > 0 is assumed from the start, and we can use the condition Re[*λ*_1_] → 0^+^ to determine the critical coupling strength for the synchronization transition, which leads to
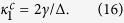
2. This concise result is significant: the critical coupling strength is explicitly determined by the properties of the Lorentzian frequency distribution: namely, it is proportional to the width, but inversely proportional to the central frequency. From Eq. (15), we can also infer that such a solution is valid only for the case Δ > 0. For Δ ≤ 0, indeed, Re[*λ*_1_] < 0, which contradicts the assumption *a* > 0.
3. *a* = 0. In this case, *λ* = *ib*. *f*(*ω*) has one pole *ω*_1_ = −*b* on the real axis, and two poles *ω*_2_ = Δ + *iγ* and *ω*_3_ = Δ − *iγ* in the upper and lower complex plane, respectively. We can choose a contour in the upper half complex plane, but bypassing the pole on the real axis. Accordingly, Eq. (12) becomes:
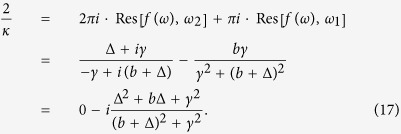
Apparently, the solution for this equation is 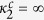 regardless of Δ. So this solution is physically unrealizable and should be neglected. By the way, from the above equation the eigenvalue can also be explicitly solved as
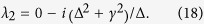
*λ*_2_ is then on the imaginary axis, consistently with the assumption *a* = 0.
4. *a* < 0. In this case, *f*(*ω*) has one pole *ω*_1_ = Δ + *iγ* in the upper complex plane; and two poles *ω*_2_ = Δ − *iγ* and *ω*_3_ = −*b* + *ia* in the lower complex plane. The integral in Eq. (12) can be conveniently done by choosing an integral contour in the upper complex plane. Accordingly, Eq. (12) becomes:

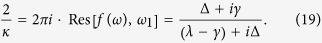


From this equation, one can obtain the eigenvalue as


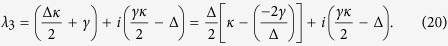


Notice that, as *a* < 0 has been assumed, one can determine the critical coupling strength by setting Re[*λ*_3_] → 0^−^, i.e.,


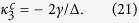


For Δ ≥ 0, the solution of transition point is physically unreasonable. Nevertheless, for Δ < 0, 
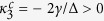
 is significant.

**Figure 1:**
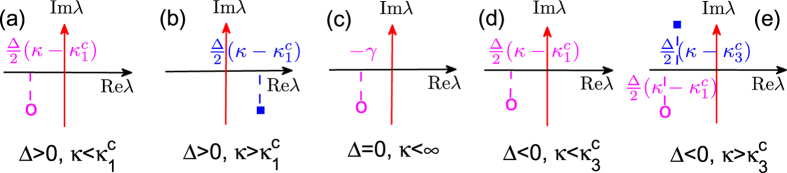
The spectra for the characteristic equation (9). For a Lorentzian frequency distribution, the continuous spectrum is the whole imaginary axis. The solid squares denote, instead, the discrete eigenvalues. The purple ovals in (**a**,**c**–**e**) mark the *ghost* (fake) eigenvalues predicted by the linear theory, which are actually valid only on the right half complex plane. As discussed in the text, such ghost eigenvalues remarkably control the decaying rate of order parameter *r*(*t*) in the neutrally stable regimes, exactly as in the Landau damping context. It should be pointed out that the *ghost* eigenvalue in (**d**,**e**) could also be above the real axis, depending on parameters. For better visualization, we do not plot the other irrelevant (fake) eigenvalues.

Based on the above analysis, one can extract the critical coupling strength *κ*^*c*^ as well as determine the stability properties of the incoherent state, which are schematically reported in Fig. 1. Specifically:

(Case 1) Δ > 0: the conformists prevail over the contrarians. 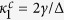 and the spectra are schematically plotted in Fig. 1(a,b). When 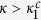, Eq. (9) has a continuous spectrum on the imaginary axis and a discrete eigenvalue in the right half complex plane. Accordingly, the incoherent state is unstable. When 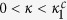, there is no discrete eigenvalue, and the incoherent state is neutrally stable.

(Case 2) Δ = 0: conformists and contrarians are equal in number. In this case, 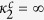, implying that synchronization can never be achieved. As shown in Fig. 1(c), here no discrete eigenvalues exist outside the imaginary axis, and therefore the incoherent solution is always (i.e. for any arbitrary coupling strength) neutrally stable.

(Case 3) Δ < 0: the contrarians prevail over the conformists. 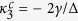, and the spectra are shown in Fig. 1(d,e). Besides the continuous spectrum on the imaginary axis, there is no discrete eigenvalue when 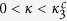, while there is an eigenvalue in the left half complex plane when 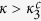. So in this case, the incoherent solution is also always neutrally stable.

The current situation shares connections and differences with the classical Kuramoto model^7^,^8^. For Δ > 0, the stability of the incoherent state is the same as that of refs 7,8, though the equations of the two models are essentially different. Nevertheless, for Δ = 0 and Δ < 0, the incoherent state is always neutrally stable, regardless of *κ*. The two latter phenomena are novel, and inherent in our frequency-weighted Kuramoto model, which allows the two populations of conformist and contrarian oscillators to coexist.

### The Landau damping

In order to unveil LD in the model, we analytically extract the equation ruling the relaxation behavior of *r*(*t*) in all four neutrally stable regimes predicted by the linear theory, i.e., the cases corresponding to Fig. 1(a,c–e). As it will appear momentarily, it is found that *r*(*t*) decays, indeed, exponentially in all cases in which the incoherent state is neutrally stable. Remarkably, the decaying rate can be analytically predicted, as follows. Combining Eq. (6) and Eq. (8) leads to *r*(*t*) = 2*πε*|*R*(*t*)|, where


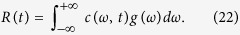


Then, Eq. (9) becomes


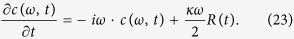


For any given initial condition *c*_0_(*ω*) = *c*(0, *ω*), the solution of Eq. (23) is:


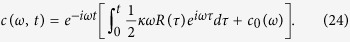


In a self-consistency way, substitution of Eq. (24) into Eq. (22) leads to


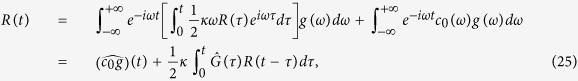


where the hat denotes the Fourier transform, i.e., 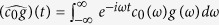 and 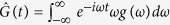.

To solve this integral equation, we apply the Laplace transform and get


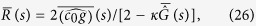


where the bar denotes here the Laplace transform 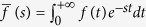. By choosing as initial condition *c*_0_(*ω*) = 1, we have


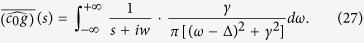


Note that for Laplace transform, Re[*s*] > 0 can always be satisfied. So the integrand in the above equation has only one pole in the lower half complex plane. A contour integral in the lower half complex plane conveniently gives


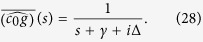


Similarly,


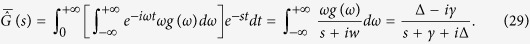


Substitution of the above two equations into Eq. (29) and application of the inverse Laplace transform lead to


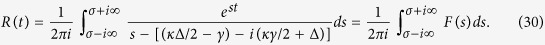


Now, the only pole *w*_*s*_ = (*κ*Δ/2 − *γ*) − *i*(*κγ*/2 + Δ) is on the left of line Re[*s*] = *σ* for sufficiently large *σ* (that is guaranteed in the Laplace transform), and therefore one can apply residue theorem along the contour on the left of the line Re[*s*] = *σ* in the complex plane. This finally gives


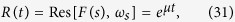


where the decaying exponent is


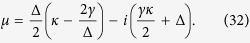


Interestingly, we find that *μ* = *λ*_1_ by comparing Eq. (32) with Eq. (15). Notice that *λ*_1_ is valid only on the right half complex plane, i.e., when *a* > 0. In the neutrally stable regimes (as shown in Fig. 1, *λ*_1_ is not supposed to hold. In such a sense, we say that *λ*_1_ is the *ghost* (fake) eigenvalue that actually controls the decaying of *r*(*t*). Mathematically, Eq. (31) can be obtained from Eq. (30) by application of analytic continuation for the integrand, which can lead to a pole *s* = *μ* in the left half complex plane. Such a latter pole is the fake eigenvalue that is responsible for the exponential decay of the order parameter. Notice that, in the derivation of Eq. (32), we do not yet imposed specific constraints to the functional form of *μ*, therefore [based on the closed form of *R*(*t*)], we here below summarize what happens to the relaxation behavior of *r*(*t*) in the three different cases of Δ.

(Case 1) Δ > 0. One obtains that the critical coupling strength is 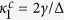, by setting Re[*μ*] = 0. When 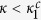 [where the linear theory predicts neutral stability, Fig. 1(a)], *r*(*t*) decays exponentially with Re[*μ*] = Δ*κ*/2 − *γ* = Re[*λ*_1_]. Therefore, it is remarkable that the damping rate is actually determined by the value of the *ghost* eigenvalue *λ*_1_ in Fig. 1(a), exactly like what happens in the LD mechanism.

(Case 2) Δ = 0. The incoherent state is neutrally stable regardless of *κ* [Fig. 1(c)]. According to Eq. (32), *r*(*t*) *always* decays exponentially with Re[*μ*] = −*γ* = Re[*λ*_1_], once again like if the *ghost* eigenvalue *λ*_1_ in Fig. 1(c) would still be present.

(Case 3) Δ < 0. From Eq. (32), Re[*μ*] = Δ*κ*/2 − *γ*, which is *always* negative. Therefore, *r*(*t*) does decay exponentially for any value of *κ*, corresponding to the neutrally stable regimes predicted by linear theory [Fig. 1(d,e)]. Remarkably, once again the decaying rate Re[*μ*] = Δ*κ*/2 − *γ* = Re[*λ*_1_] rather than Re[*λ*_3_] even when it is negative [Fig. 1(e)]! To summarize, in all the regimes of neutral stability, as shown in Fig. 1, it is the *ghost* eigenvalue *λ*_1_ actually controls the decaying of *r*(*t*).

In the above, focusing on the typical Lorentzian FD, we are able to analytically illuminate the LD effects in model (1). How about other FDs? This is an important issue regarding the general validity of LD in our model. Actually, we have considered several other FDs, such as uniform, Gaussian, and triangle, etc. Although the complete analytical treatment turns out to be difficult, we still can get some general qualitative results based on our above study on the Lorentzian FD. In fact, the linearized characteristic equation, i.e., Eq. (9), is generally valid regardless of FD. Due to the continuous spectra on the imaginary axis, as long as the discrete spectra are on the left complex plane, i.e., below the synchronization threshold, the incoherent state of the system must be neutrally stable. In other words, the incoherent state is generally excluded to be linearly stable in our model for any FDs. Then how does the order parameter behave in such regime? To this end, we turn to numerical simulations and the results will be reported in the following section.

### The numerical simulations

**Figure 2:**
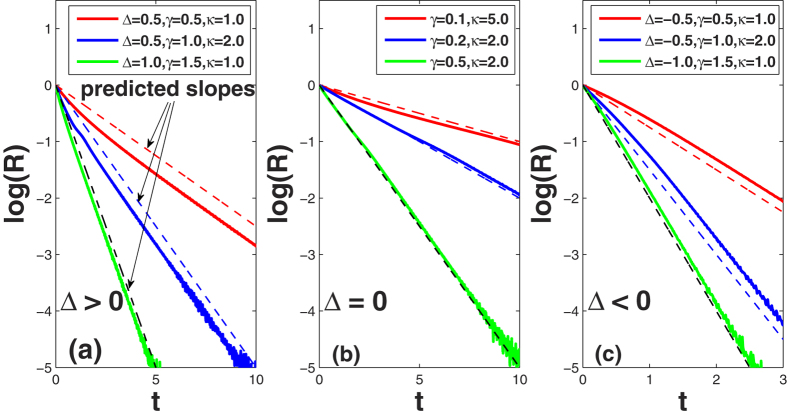
Landau damping effects in the order parameter *R*(*t*) with Lorentzian FD. Horizontal lines are time, solid lines refer to the numerical solutions of Eq. (1), and dashed lines are the analytical predictions of Eq. (32). The three log-linear plots refer to the cases Δ > 0 (**a**), Δ = 0 (**b**), **a**nd Δ < 0 (**c**). All curves belong to the neutrally stable regime of the incoherent state predicted by linear theory. In the numerical simulations, the initial states of the system are set in the fully coherent states.

**Figure 3:**
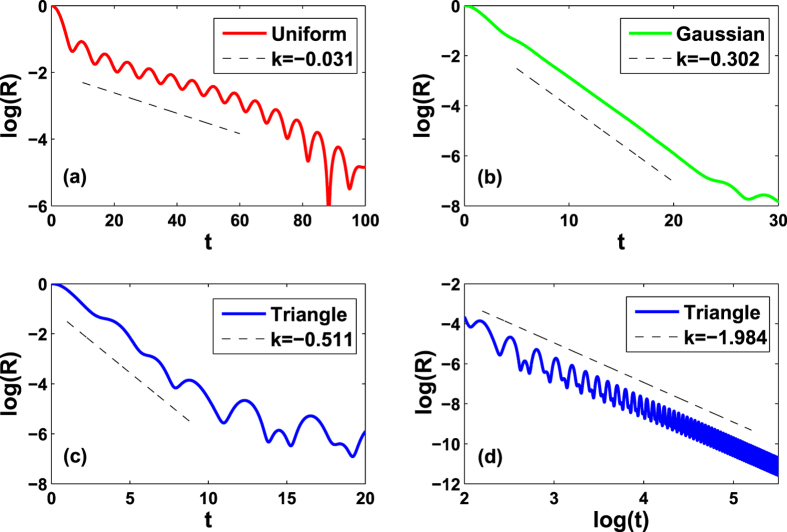
Landau damping effects in the order parameter *R*(*t*) with uniform, Gaussian, and triangle FDs. Horizontal lines are time, solid lines refer to the direct numerical solutions of Eq. (1) with *κ* = 1.0, and dashed lines are the fitted straight lines with slope *k*. Note that (**a**–**c**) **a**re semi-log plots, while (**d**) is double-log. To effectively suppress the fluctuation of the order parameter, the number of oscillator *N* = 25600000. The formulae and parameters for the three FDs are as follows. Uniform: *g*(*ω*) = 1 for |*ω* − Δ| < 0.5, and 0 otherwise. Δ = 0.1. Gaussian: 
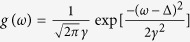
 with Δ = 0.1 and *γ* = 0.5. Triangle: *g*(*ω*) = (*γ* − |*ω* − Δ|)/*γ*^2^ for |*ω* − Δ| < *γ*, and 0 otherwise. Δ = 0.1 and *γ* = 0.5.

Finally, we compare our analytical predictions with direct numerical simulations of Eq. (1). We first verify that the numerics match all theoretical predictions, as shown in Fig. 2, and confirm an exponential relaxation of *R*(*t*) in the regimes of neutral stability, for all the three cases of Δ when the FD is chosen as Lorentzian. Then we have conducted direct numerical simulations for other typical FDs. The results are shown in Fig. 3. It is found that the order parameters typically decay in the neutrally stable regimes with uniform, Gaussian, and triangle FD. In the short term, the decay is exponential. In the long term, the decay could be power-law. This is similar to the situation reported in ref. 5. The above numerical results suggests that the LD effects might be a generic phenomenon in model (1). It is a challenging task for us to theoretically predict the decay rates for these FDs in the future.

**Figure 4:**
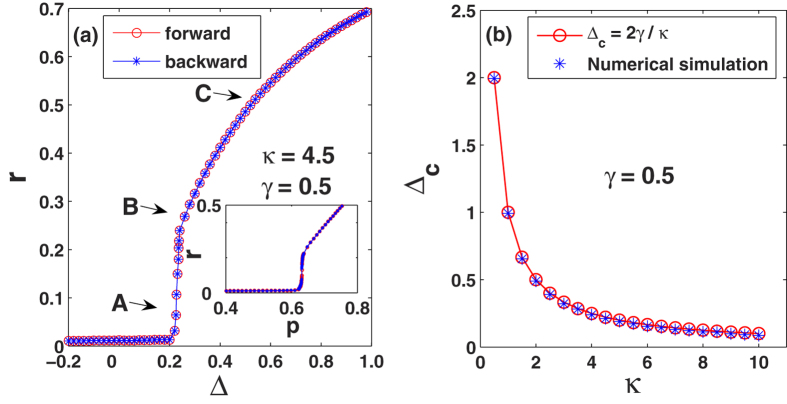
Synchronization transition in Eq. (1) in the presence of conformist and contrarian oscillators. (**a**) *r* vs Δ (see text for definitions). Letters A, B, C denotes the three conditions that will be analyzed in the next Figure. The inset reports *r* vs *p*, i.e., the proportion of conformist oscillators in the ensemble, which (for any given *γ*) is entirely controlled by Δ. (**b**) Given *γ*, Δ_*c*_ comes out to be inversely proportional to *κ*.

Next, starting from random initial conditions for the phases, we gradually step up (forward transition) and down (backward transition) Δ from (and to) a negative initial value (Δ = −0.2), while keeping *γ* = 0.5 and *κ* = 4.5 constant, this way piecemeal modulating the proportion of conformist oscillators in the ensemble. Results are shown in Fig. 4(a), where it is seen that a continuous (fully reversible) synchronization transition occurs at the critical point Δ_*c*_ = 2*γ*/*κ*. In Fig. 4(b), Δ_*c*_ vs *κ* is plotted, and numerical results perfectly verify the prediction of an inverse proportion in the critical point. On its turn, this means that, as long as the number of the conformists prevails over that of the contrarians, synchronization will always occur for a large enough coupling strength.

**Figure 5:**
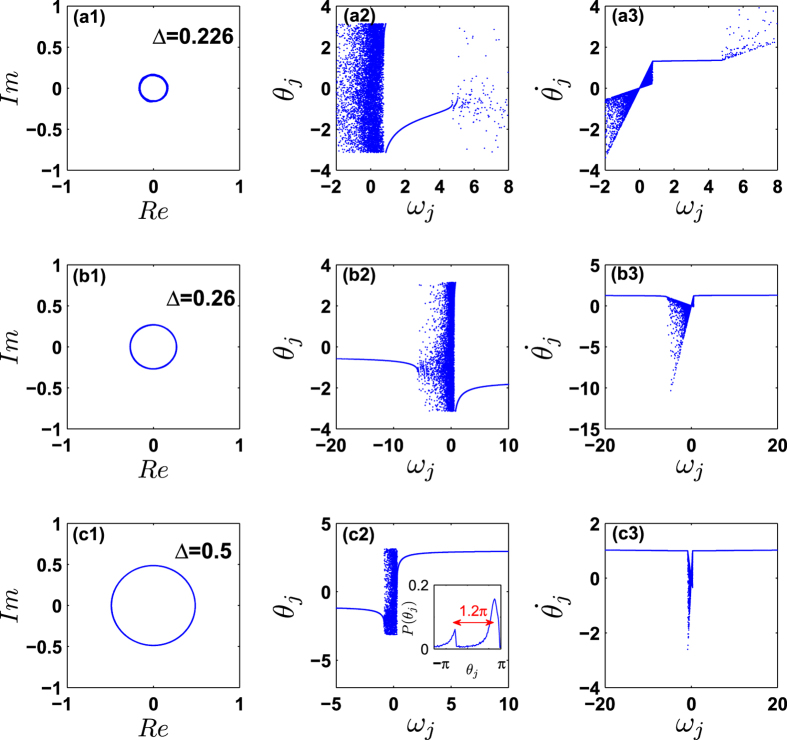
Characterization of the coherent states. *κ* = 4.5 and *γ* = 0.5. Rows (**a**–**c**) correspond to Δ = 0.226, 0.26, and 0.5, respectively, i.e., to the points A, B, and C of Fig. 4(a). Column 1 plots the order parameter in the complex plane after a transient stage, while columns 2–3 correspond to the snapshots of the distributions of the instantaneous phases and the frequencies at *t* = 2,500. In (**a**), the conformists slightly prevail over the contrarians, and only a small part of conformists form a coherent cluster, rotating at a certain frequency along the unit circle. In (**b**), the coherent cluster of conformists continuously expands, while the contrarians begin forming a coherent cluster. Both clusters rotate at the same frequency along the unit circle, with a constant phase difference between them. In (**c**) more conformists are present in the system. The cluster of the conformists further enlarges, leading to the increase of the order parameter. The inset in (c2) shows the phase distribution at that moment. The phase difference between two peaks is 1.2*π*.

Going further, one can even unveil the mechanisms and processes taking place during the path to synchronization. Namely, Fig. 5 reports the instantaneous distributions of phases and frequencies that characterize the coherent states. As Δ is below the critical value, synchronization cannot be achieved. When Δ exceeds Δ_*c*_, a phase-locking cluster of conformists (*ω*_*i*_ > 0) first appears [as shown in Fig. 5(a)], without an associated cluster of synchronized contrarians. As Δ increases, more and more conformists join the synchronized cluster. Only at an intermediate moment, the contrarians start forming a synchronized cluster, as shown in Fig. 5(b). This latter fact is actually striking, as it demonstrates that in such ensembles the synchronization of contrarians can only be achieved *after* the synchronized cluster of conformists is large enough, as if the former process would be actually induced by the latter. By further increasing Δ, the cluster of conformists becomes larger and larger by recruiting more and more drifting oscillators, which leads to larger order parameter, as shown in Fig. 5(c). The two clusters (that of conformist and that of contrarians) rotate with the same frequency along the unit circle. During the rotation, they are relatively static with each other, as shown in the inset of Fig. 5(c2), i.e. the two peaks in the phase distribution keep a constant difference 1.2*π* (or 0.8*π*). As a consequence, the system enters (after synchronization) into a traveling wave state, which however (and generally) is not the *π* state, i.e. that state where the phase difference between the two clusters is always *π*^15^.

**Figure 6:**
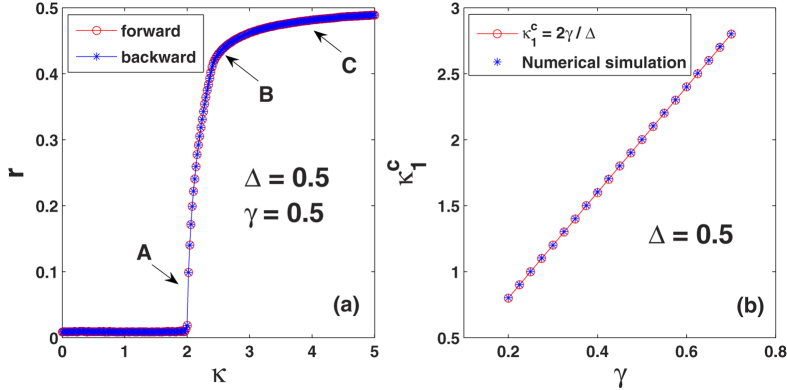
Characterization of synchronization in Eq. (1) as the coupling strength increases. The FD is fixed as Δ = 0.5 and *γ* = 0.5. (**a**) The order parameter *r* vs the coupling strength *κ*. Letters A, B, C denotes the three conditions that will be analyzed in the next Figure. (**b**) Given fixed central frequency of FD Δ = 0.5, the critical values of 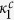 vs the width of FD *γ*.

Finally, we study how the coupling strength affects the synchronization in Eq. (1) when the ratio between conformists and contrarians is fixed. To this end, we keep Δ = 0.5 and *γ* = 0.5, and compute the order parameter with respect to the coupling strength. As shown in Fig. 6(a), the system also exhibits a second-order transition to synchronization as the coupling strength increases. The theoretical critical point has been analytically given in Eq. (16). To verify this, we numerically computed the critical point 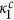 with different *γ* while keeping Δ = 0.5 as a constant. As shown in Fig. 6(b), as the width of the FD increases, the critical point 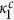 increases linearly. Once again, the numerical data perfectly coincide with the theoretical predictions. Physically, Fig. 6(b) suggests that, given the central frequency of FD, the system becomes more and more difficult to be synchronized as the width of the FD increases. The result can be heuristically understood as follows. For our model, the ratio between the conformists and the contrarians plays a crucial role in determining whether synchronization can be achieved or not. Both Δ and *γ* can affect this ratio. According to Eq. (4), the proportion of conformists in the system can be analytically given as 
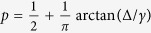
. Therefore, given Δ, *p* will decrease as *γ* increases. This explains why larger coupling strength is required to achieve synchronization when *γ* increases.

**Figure 7:**
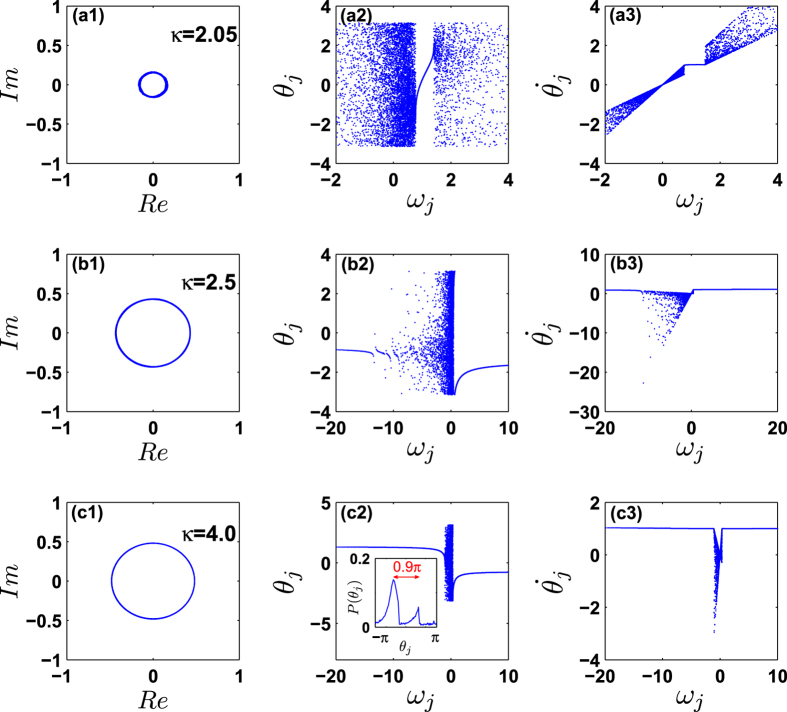
Characterization of the coherent states. Δ = 0.5 and *γ* = 0.5. Rows (**a**–**c**) correspond to *κ* = 2.05, 2.5, and 4.0, respectively, i.e., to the points A, B, and C of Fig. 6(a). The arrangement of panels are the same as in Fig. 5.

Similarly, we can characterize the microscopic properties of the coherent states after achieving synchronization in Fig. 6(a). The results are shown in Fig. 7. It is shown that when the FD is fixed, i.e., Δ > 0 and *γ* are given, the system first forms a cluster of conformists as the coupling strength exceeds the critical point, as shown in Fig. 7(a). Then as the coupling strength increases further, this cluster grows larger and simultaneously a cluster of contrarians begins to form, as shown in Fig. 7(b,c). Through extensive simulations, we have verified that the coherent states in this situation are always traveling wave states. As shown in Fig. 7(c2), generally they are not the *π* state^15^.

It has been shown that the traveling wave states usually occur when the symmetry in either the natural frequencies or the coupling strength itself is broken^20^. In our study, the FD is generally asymmetric as long as Δ ≠ 0. As a consequence, the traveling wave solutions should be typical. Furthermore, the occurrence of traveling wave states can be heuristically understood as follows. As pointed out in ref. 21, the phase-locking condition for a pair of oscillators in system (1) is


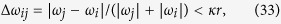


i.e. a pair of oscillators is forbidden to synchronize with each other (for any given *κ*) if such a condition does not hold. Now, if a pair of oscillators have natural frequencies with different signs, Δ*ω*_*ij*_ = 1. In the incoherent state, *κr* is typically much less than 1, and therefore those oscillator pairs with Δ*ω*_*ij*_ = 1 will most likely violate the above condition. At variance, oscillator pairs with the same frequency sign are easier to synchronize, especially when their natural frequencies are close enough. This explains why clusters always form among conformists or contrarians, as observed in Fig. 5. In the traveling wave state, both coherent clusters of conformists and contrarians rotate with the same instantaneous frequency that is greater than 0. According to Eq. (2), the conformists turn to approach the mean-field phase *ϕ* due to *ω*_*i*_ > 0. Nevertheless, in order to make the instantaneous frequency 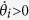 of contrarians, sin(*ϕ* − *θ*) must be less than 0, i.e. their phases will always rotate against the mean-field phase *ϕ*, and one will observe a phase difference between the two clusters (conformists and contrarians) which is always greater than *π*/2.

Previously, ref. 15 has investigated a system with both conformists and contrarians. However, the model in ref. 15 is essentially different from ours in terms of both the frequency distribution and the coupling scheme. In ref. 15, the natural frequencies satisfy the Lorentzian distribution, but the coupling is chosen as double *δ* function, i.e., the coupling strength takes either *κ*_1_ < 0 or *κ*_2_ > 0. In such way, the conformists and the contrarians are defined. Since ref. 15 considered Lorentzian distribution that is symmetric with respect to 0, it is reasonable that the *π* states, in which the average frequency of oscillators is 0, are observed (besides the traveling wave states). In our model, both the distribution of natural frequencies and the couplings are *asymmetric* Lorentzian, so generally the average frequency is not 0^20^. As a consequence, the traveling wave states are typically observed as described above.

## Discussion

Two decades ago, Strogatz *et al.* reported LD effects in the classical Kuramoto model^5^. In this work, we extended the formalism to a frequency-weighted case, where conformist and contrarian oscillators interact. The used analytical treatments, such as linear stability analysis and the method of Laplace transform, are inherited from ref. 5, but our model substantially differs from past approaches in the following aspects. On the one hand, the frequency-weights of our model lead to heterogeneous couplings that are essentially different from the homogeneous couplings in the classical Kuramoto model. On the other hand, the frequency-weighted coupling distinguishes two types of oscillators in the system, i.e., the conformists and contrarians, according to the signs of natural frequencies. Moreover, our model exhibits certain new features. For instance, as shown in Fig. 1(e), the system is neutrally stable though it has a discrete eigenvalue on the left complex plane. Remarkably, it is the *ghost* eigenvalue (rather than this *alive* one) that actually controls the decay of the order parameter.

Recently, the Ott-Antonsen (OA) ansatz has been proposed to obtain the low dimensional dynamics of a large system of coupled oscillators^22^, and has been successfully applied in many situations^15^. It turns out that such a method can be used to analyze the stability of the incoherent state (to predict the critical coupling strength and the decaying rate of the order parameter). However, it fails to treat properly the partially coherent state in our case. Therefore, the OA method does not provide information more substantial than the traditional linear stability analysis. As the latter can also provide detailed insights on eigen-spectra (that are indeed crucial in the context of LD), we preferred to make use of it in the present work.

Together with providing evidence of LD effects in the synchronization of coupled oscillators, our results are of significance in that they contribute to shed light on the mechanisms at the basis of some phenomena beheld in social and economical sciences. For example, in western countries, the mainstream politics is the multiparty system. Basically, there exist both competition and cooperation among the multi parties. Nevertheless, as far as a specific political issue be concerned (mean-field), some parties tend to agree (conformists) while the others tend to oppose (contrarians). As a consequence, we frequently observe the phenomenon that “the left wing” confront with “the right wing” in the congress. In addition, the weaker side becomes more united as the stronger sides becomes more powerful, while the two sides hardly compromise with each other. Similar phenomena can also be found in economical systems. For example, the Intel microprocessor is dominant in PC market over the years (mean-field). As a consequence, the numerous PC producers in China face two choices: either use it (conformists), or use others such as AMD microprocessor (contrarians). This leads to the polarization of PC producers in China: Dell, TCL, Haier, and Founder, etc, vs Legend, HP, and Shenzhou, etc. Our results may then enlighten the reasons for the occurrence of such circumstances.

In summary, we investigated the synchronized dynamics of an ensemble of phase oscillators when the coupling to the mean field is frequency-weighted and two populations of oscillators (conformists and contrarians) can be identified. Analytically, we derived the critical coupling strength for synchronization and determined the stability of the incoherent state. In all regimes where the linear theory predicts neutral stability, the order parameter decays exponentially, in analogy with the Landau damping effect in plasma physics. Extensive numerical simulations fully support the theoretical predictions, and show that a continuous synchronization transition occurs by changing the ratio of conformists and contrarians. Traveling wave states are generically observed after synchronization. This work is helpful to enhance our understandings of LD effects in coupled oscillators systems.

## Additional Information

**How to cite this article**: Qiu, T. *et al.* *Landau damping* effects in the synchronization of conformist and contrarian oscillators. *Sci. Rep.* **5**, 18235; doi: 10.1038/srep18235 (2015).
